# Effects of repetitive transcranial magnetic stimulation at different targets on brain function in stroke patients: a randomized controlled trial

**DOI:** 10.3389/fneur.2024.1454220

**Published:** 2024-09-30

**Authors:** Li Zhao, Li Chen, Chunyan Wang, Sha Li, Chunxiao Wan

**Affiliations:** ^1^Department of Rehabilitation, Tianjin Medical University General Hospital, Tianjin, China; ^2^Department of Imaging, Tianjin Medical University General Hospital, Tianjin, China

**Keywords:** ischemic stroke, rTMS, SMA, rehabilitation, neural remodeling

## Abstract

**Introduction:**

Repetitive transcranial magnetic stimulation (rTMS) can improve post stroke motor function. However, there is little research on targets. The purpose of this study is to investigate the effects of rTMS therapy with different targets on post stroke motor function and neural plasticity.

**Methods:**

Fifty-four subjects were randomly divided into M1 (Primary motor area) group, SMA (supplementary motor area) group and Sham group, and were given 10 Hz on the affected M1 area, SMA area and sham stimulation rTMS. The primary outcomes included Fugl-Meyer Assessment Upper Extremity Scale (FMA-UE), Fugl-Meyer Assessment Lower Extremity Scale (FMA-LE) and Berg balance scale (BBS). Secondary outcomes: amplitude of low frequency fluctuation (ALFF), regional homogeneity (ReHo) and functional connectivity (FC) were analyzed by functional magnetic resonance imaging (fMRI) to evaluate brain functional activation and functional connectivity changes.

**Results:**

The 2-way repeated-measures ANOVA revealed a significant group × time interaction (*F* = 23.494, *p* < 0.001; *F* = 10.801, *p* < 0.001; *F* = 17.812, *p* < 0.001) in the FMA-UE, FMA-LE and BBS scores. *Post hoc* analysis indicated that 4 weeks of SMA rTMS resulted in an increase in FMA-UE, FMA-LE and BBS scores compared with Sham group (*p* = 0.006; *p* = 0.033; *p* = 0.012), SMA group was significantly increased in BBS compared with M1 group (*p* = 0.034). Moreover, there were significant effects of time in all 3 groups in the FMA-UE, FMA-LE and BBS scores (*p* < 0.001). In addition, the increase of ALFF in the supramarginal gyrus on the affected side was correlated with better FMA-UE recovery, the increase of ALFF in the middle temporal gyrus and the middle frontal gyrus on the affected side was positively correlated with the improvement of BBS, and the ALFF in the cerebellum on the healthy side was negatively correlated with the improvement of BBS. There was a positive correlation between FC (SMA – ipsilateral cerebellum) changes and BBS changes in SMA group.

**Discussion:**

In conclusion, SMA-rTMS intervention has a better recovery effect on motor dysfunction after stroke than Sham-rTMS. SMA-rTMS led to similar improvement on motor function but significantly greater improvement on balance compared to M1-rTMS, and this may pave a new way for stroke rehabilitation.

**Clinical trial registration:**

Registration number: ChiCTR2200060955, https://www.chictr.org.cn/.

## 1 Introduction

Stroke has become the main cause of morbidity and mortality and is showing a trend toward younger ages of onset (1, 2). Approximately 70 to 80% of patients with stroke experience varying degrees of functional impairment, with 80% of patients experiencing motor dysfunction, which is the main cause of disability (3). Research shows that the rate of cerebral nerve tissue repair is the fastest from a few hours to approximately 10 weeks after stroke and then gradually slows down and reaches a plateau 3 months after stroke (4, 5). The early stage of subacute phase is the golden period for functional recovery, and most clinical rehabilitation patients are also in this stage. Therefore, the first step is to clarify the changes in brain function of patients in this stage, which is the basis for subsequent interventions. Personalized rehabilitation interventions (including physical and occupational therapy) are the gold standard for promoting poststroke motor function recovery (2).

The nervous system forms a large and complex neural network through self-regulation and repair. The neural network can continuously undergo functional reorganization through external stimuli or intrinsic regulation, triggering a series of cellular and other biological processes after stroke, leading not only to necrosis but also to synaptogenesis and axon sprouting (6), laying the foundation for rehabilitation. Recently, transcranial magnetic stimulation (TMS) has become a new safe method for regulating cortical excitability, improving motor function in stroke patients by increasing motor output. As one of the modes, rTMS regulates neural plasticity and promotes neural repair by modulating the excitability and neurobiological function of the cerebral cortex. It has obtained “A-level evidence” in the neurorehabilitation application of motor function after stroke (7). However, there is still a lack of evidence for targeted stratification and individual treatment. In previous studies, M1 and cerebellum were the main targets for treating post-stroke motor function (8–10). The supplementary motor cortex (SMA), as an important motor related brain area, plays an important role in movement preparation, control and coordination (11), and is involved in continuous movement pattern programming, connecting primary motor cortex and spinal cord, and participating in movement execution (12). Previous studies have found that SMA plays an important role in the recovery of balance function and gait after stroke (13, 14). However, few studies have considered SMA as the target of rTMS (15), especially in the systematic comparison between M1 rTMS and SMA rTMS.

In recent years, the widespread application of functional magnetic resonance imaging (fMRI) has further advanced clinical research on brain function. As a non-invasive brain imaging method, it can detect the functional connections between various brain regions connected by synapses, and detect the plasticity between networks (11), which is expected to become a biomarker for precision medicine. In this study, we measured three rs-fMRI-based markers to predict local brain neural activity and functional connectivity; these measures comprised amplitude of low-frequency fluctuation (ALFF), regional homogeneity (ReHo), and functional connectivity (FC).

In condition, previous studies have shown that rTMS is helpful in improving motor dysfunction after stroke (16–18), but the possible mechanisms of stimulation parameters, targets, and neural remodeling are still unclear (19–21). In this study, based on the classic “interhemispheric competition model”, we administered high-frequency stimulation of 10 Hz to the affected side to correct the imbalance of excitability between hemispheres and promote brain function remodeling. The focus of this study is to use fMRI to assess the differences between M1 and SMA as different stimulation targets, aiming to identify more precise and optimized action targets, further optimize rehabilitation treatment plans, and refine rehabilitation treatment.

## 2 Methods

### 2.1 Study population

In this randomized controlled trial, 54 patients with cerebral infarction admitted to the Department of Rehabilitation Medicine of Tianjin Medical University General Hospital from June 27, 2022, to June 30, 2023, were selected as the study subjects. The inclusion criteria were as follows: ① Patients with subcortical ischemic stroke who met the diagnostic criteria specified in the “Key Diagnostic Points for Various Major Cerebrovascular Diseases in China 2019” formulated by the Neurology Branch of the Chinese Medical Association and have been confirmed by head spiral CT or head magnetic resonance imaging; ② First stroke, with motor dysfunction (FMA-UE<32, FMA-LE<18, BBS<20); ③ Course of disease: 2–12 weeks; ④ Right-handed; ⑤ No gender restrictions, age: 40–80 years; ⑥ Stable vital signs, clear consciousness; ⑦ MMSE (mini-mental state examination) > 21 points; and ⑧ Signed informed consent.

The exclusion criteria (15) were as follows: ① Unstable condition and secondary stroke; ② Previous peripheral nerve injury or peripheral neuropathy on the affected side; ③ Previous individua history or family history of epilepsy; ④ Severe anxiety or depression symptoms requiring control with medication; ⑤ Severe heart, lung, liver, kidney or other organ diseases; ⑥ Presence of a pacemaker, cochlear implant, or skull repair plate in the body; and ⑦ Claustrophobia.

The discontinuation and dropout criteria were as follows: ① Progressive worsening of condition or other newly diagnosed diseases rendering the patient unable to continue treatment; ② Severe adverse reactions or inability to tolerate rTMS during treatment; and ③ Requests by subjects to withdraw for their own reasons.

All participants provided written informed consent according to the Declaration of Helsinki before the experiment, as approved by the ethics committee of the hospital (approval number: IRB2022-YX-054-01), and registered in the Chinese Clinical Trial Registry (registration number: ChiCTR2200060955 at 2022-06-14).

#### 2.1.1 Trial inclusion

**Figure 1:**
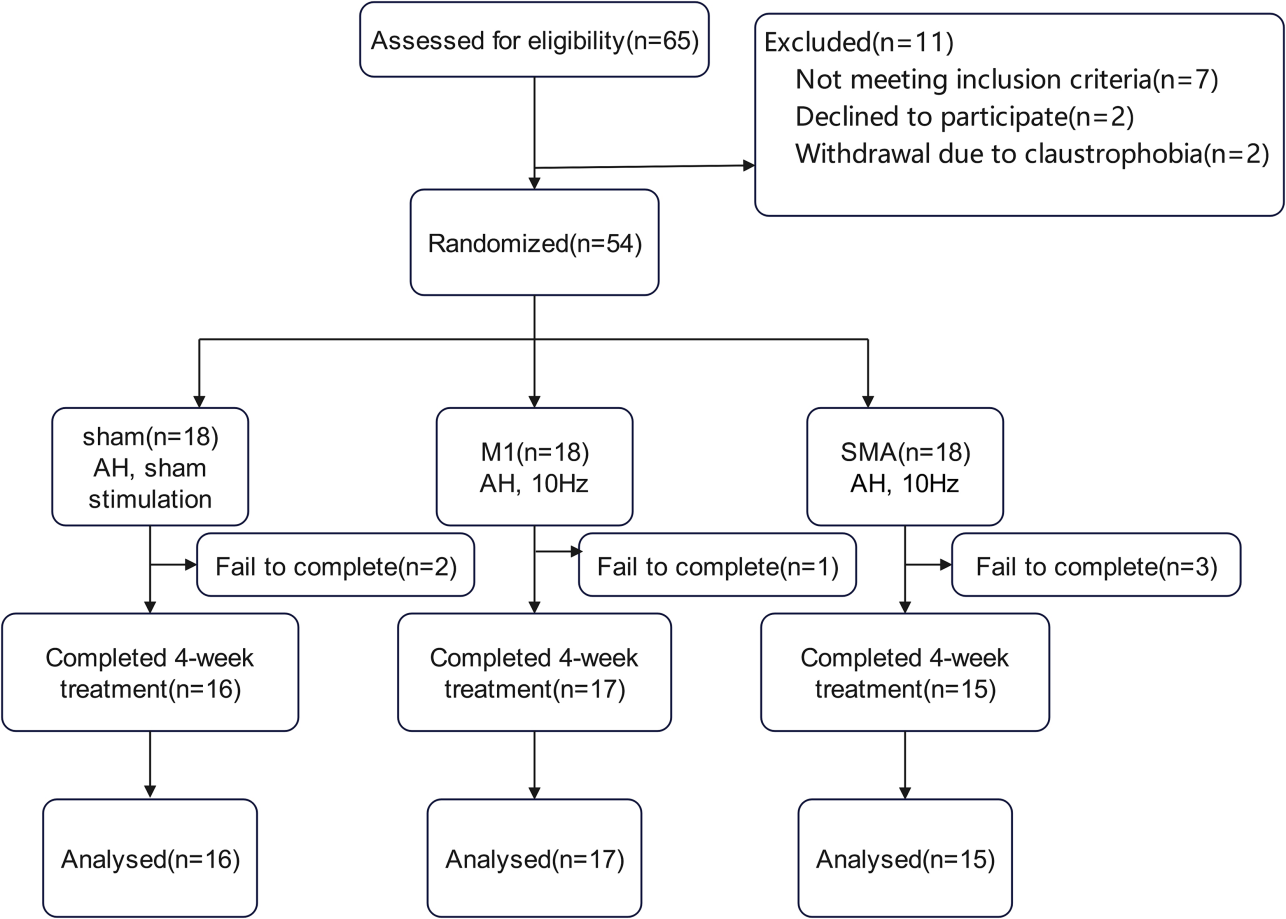
Trial flow chart.

Due to the epidemic, there were 2, 1, and 3 subjects in the three groups who did not complete the entire study process. Finally, 16 cases, 17 cases, and 15 cases from the three groups completed this study (Figure 1).

### 2.2 Research design

#### 2.2.1 Randomization and blinding

After enrollment, a simple randomization method was used, and the sample size was input into a simple statistical software program to obtain a random number table. According to the order in which the participants entered the experiment and the corresponding groups of characters in the random number table, they were randomly divided into the M1 group, SMA group, and Sham group in a 1:1:1 ratio. The intervention was transferred to the ipsilateral M1 (10 Hz) or SMA (10 Hz), or sham stimulation, respectively. The results of randomization were kept in randomly arranged opaque envelopes. Outcome measures were assessed by a physical therapist who was unaware of patient assignments. The therapists who performed physical therapy were also blinded. However, the therapist operating the magnetic stimulator was not blinded.

All three groups received the same routine rehabilitation training, including acupuncture, PT, and OT. Each rTMS intervention was preceded by routine rehabilitation as described above.

#### 2.2.2 rTMS protocol determination of simulation regions

The intervention was provided by a physiotherapist who was well trained in TMS. All subjects received rTMS treatment or sham stimulation therapy using a Mag TD 100 Hz magnetic field stimulator (YIRUIDE Medical Co., Wuhan, China). Before the first treatment, determine the resting motion threshold (RMT) of the healthy abductor pollicis brevis muscle (22, 23): the minimum stimulation intensity that causes slight contraction of the healthy abductor pollicis brevis muscle in 5 out of 10 single pulse transcranial magnetic stimulation in the brain (24). Using the 10–10 international EEG standard to locate stimulation targets (M1: midpoint of the line connecting CPz to C3 or C4; SMA: FC1 or FC2; Sham: Midpoint of Pz and C3/C4) (25). During treatment, the subjects were placed in a comfortable sitting or supine position, and a “8 coil” was placed on the stimulation target. During treatment, the coil was tangent to the surface of the skull, and the parameters were as follows: stimulation frequency: 10 Hz, intensity: 100% RMT, stimulation time: 2.5 s, interval time: 10 s, total duration: 20 min, total pulse: 2400, once a day, 5 days a week, lasting for 4 weeks. The sham group received rTMS sham stimulation.

### 2.3 Outcome

#### 2.3.1 Primary outcome

FMA-UE (26) assesses the motor function of the upper limbs (a total of 33 items, a total score of 66 points, and the lower the score, the more severe the injury).

FMA-LE (27) assesses the motor function of the lower limbs (a total of 17 items, a total score of 34 points, and the lower the score, the more severe the injury).

BBS (28) is used to assess activity limitations in daily life that require balance (a total of 14 items with a total score of 56, with higher scores indicating better balance. 0–20 points: poor balance and need to use a wheelchair; 21–40 points: some balance ability, walking with assistance; 41–56: good balance and able to walk independently).

#### 2.3.2 Secondary outcomes

fMRI scans were performed before and after intervention. ALFF was used to evaluate the changes in spontaneous activity of brain neurons (the higher the ALFF value, the stronger the spontaneous activity of brain neurons, and vice versa). ReHo was used to evaluate the synchronicity of neuronal activity in functional areas of the brain (the higher the ReHo value, the greater the synchronicity of neuronal activity in the brain region, and vice versa, the synchronicity tends to be disordered). FC was used to evaluate changes in functional connectivity between brain regions (the higher the FC value, the stronger the functional connectivity between brain regions, and vice versa, the weaker).

##### 2.3.2.1 MRI image acquisition and data processing

rs-fMRI data were obtained using the 3.0 T Siemens MRI scanner and 64 channel magnetic head coils. All subjects’ heads were fixed with customized foam pads to reduce head movement. High resolution gradient-echo echo-planar imaging (GRE-EPI) was used to scan functional imaging in resting-state fMRI to observe the spontaneous functional activity of the brain in the resting state and the functional connectivity between brain regions. The parameters were as follows: repetition time: 800 ms; echo time: 30 ms; flip angle: 56; FoV 100× 100 mm2; matrix size: 104 × 104; slice thickness: 1 mm; number of slices: 72; total time: 8′39″. During the functional magnetic resonance imaging scan, the subjects were instructed to relax, remain stationary, and close their eyes.

MATLAB (R2014b, MathWorks, Natick, MA, United States) software and DPARSF^1^ were used to preprocess the data. The data from the first 20 time points for each participant were removed to avoid scanner instability and allow subjects to adapt to scanner noise, and the data from the remaining 430 time points were preprocessed. Before data preprocessing, the images of lesions located in the left hemisphere were reversed to the right, with the right hemisphere uniformly defined as the affected hemisphere and the left hemisphere as the healthy hemisphere. Pretreatment included slice-timing correction, head movement correction, spatial standardization, spatial smoothing, removal of linear drift, low-frequency filtering (0.01 ~ 0.1 Hz) and removal of physiological confounding factors (head movement, whole brain signal, white matter signal and cerebrospinal fluid signal). For a given voxel, the time-dependent data were converted to the frequency domain using a fast Fourier transform. ALFF and ReHo were calculated within the range of 0.01 to 0.1 Hz for each voxel. Finally, all ALFF and ReHo images were smoothed spatially using a 4 mm full width at half maximum Gaussian kernel.

Based on the selection of the M1 and SMA of the cerebral cortex as intervention targets, to observe the changes in functional connectivity with other brain regions after intervention, we selected the left and right M1 and SMA (Supplemental material S1) as seed points for FC analysis and analyzed the correlation between seed points and total brain elements at the voxel level. Finally, the FC values obtained from the correlation analysis were normalized using the Fisher z-transform, and a z-value map of the entire brain was created.

### 2.4 Statistical analysis

This study was a randomized controlled trial, and the three groups were M1 group, SMA group, and Sham group. The FMA-UE, FMA-LE, and BBS scales were used as the primary clinical outcome indicators for sample size estimation. According to the results of the preliminary experiment, let a = 0.05, 1-*β* = 80%, One-way analysis of Variance F-Tests under the Means menu of PASS 2021 software were used to calculate the FMA-UE, FMA-LE and BBS. The minimum total sample size for the three groups was *N* = 48. Taking into account the cases of loss to follow-up and refusal to follow-up according to 10%, 48 × (1 + 0.1) = 53 cases, so a total of 54 cases were drawn up in the three groups, 18 cases in each group.

SPSS 27.0 statistical analysis software was used for data processing, and (𝑥̅±𝑠) was used for quantitative data analysis. Paired sample *t* tests were used to compare pre- and post-treatment data within groups. A 2-way repeated-measures analysis of variance was used to analyze the group × time interaction. The least significant difference (LSD) test was used for the subsequent comparison between the two groups. When *p* < 0.05, differences were considered statistically significant. SPM12 software was used to perform dual sample *t* tests, paired *t* tests, and one-way ANOVA on ALFF, ReHo, and FC values to obtain significant differences (q_FDR corr._ < 0.05). Pearson correlation analysis was used to measure the relationship between primary outcomes and imaging markers. The results were visualized using Xjview software.

## 3 Results

### 3.1 Baseline clinical characteristics and demographic data

No significant differences were found at baseline in the demographic or clinical evaluation data of the three groups of patients (*p* > 0.05) (Table 1).

**Table 1:** The general information and baseline clinical assessment.

	Sham (*n* = 18)	M1 (*n* = 18)	SMA (*n* = 18)	*F*	*p*
Age	58.111 (8.109)	57.889 (7.813)	57.778 (8.782)	0.008	0.992
Female (*n*)	7	8	9		
Duration from onset (d)	29.333 (22.077)	27.778 (19.185)	33.222 (19.185)	0.340	0.713
left/right Hemisphere (*n*)	9/9	8/10	10/8		
MMSE	23.056 (2.413)	22.778 (1.555)	23.111 (1.568)	0.161	0.852
NIHSS	11.611 (5.564)	12.667 (4.159)	13.333 (3.881)	0.643	0.530
FAM-UE	14.111 (7.128)	14.722 (4.688)	16.389 (5.215)	0.751	0.477
FMA-LE	14.167 (5.844)	15.167 (6.401)	16.056 (4.905)	0.486	0.618
BBS	12.500 (5.272)	12.500 (7.318)	12.611 (7.171)	0.002	0.998
ADL	40.833 (21.574)	32.500 (12.976)	38.333 (14.552)	1.168	0.319

MMSE, mini-mental state examination; NIHSS, the National Institutes of Health Stroke Scale; FMA-UE, Fugl-Meyer motor assessment scale-upper limb; FMA-LE, Fugl-Meyer motor assessment scale-lower limb; BBS, Berg Balance Scale; ADL, Activity of Daily Living Scale. Data are presented as mean (standard deviation).

### 3.2 Functional evaluation

**Figure 2:**
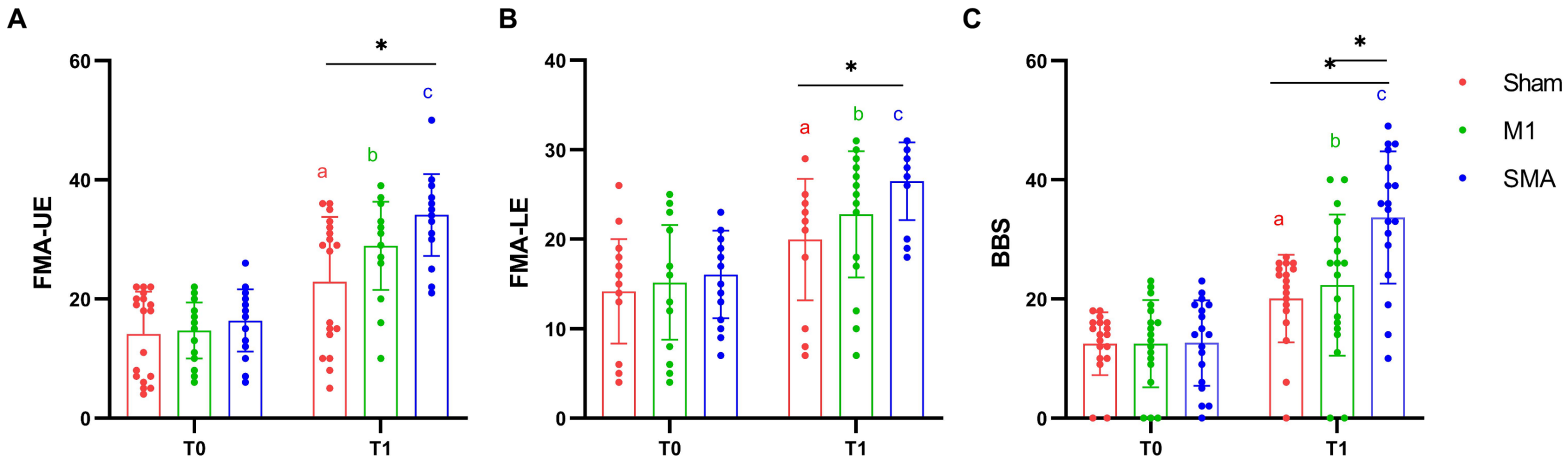
Motor function assessment. **(A)** FMA-UE assessment; **(B)** FMA-LE assessment; **(C)** BBS assessment. T0: baseline, T1:4W. *: *p* < 0.05, **: *p* < 0.01. a, b, and c: Significance of paired *t*-test before and after intervention (P_a,b,c_ < 0.001).

The 2-way repeated-measures ANOVA revealed a significant group × time interaction (*F* = 23.494, *p* < 0.001; *F* = 10.801, *p* < 0.001; *F* = 17.812, *p* < 0.001) in the FMA-UE, FMA-LE and BBS scores. *Post hoc* analysis indicated that 4 weeks of SMA rTMS resulted in an increase in FMA-UE, FMA-LE and BBS scores compared with Sham group (*p* = 0.006; *p* = 0.033; *p* = 0.012). The SMA group also showed significantly increased in BBS compared with M1 group (*p* = 0.034). Moreover, there were significant effects of time in all 3 groups in the FMA-UE, FMA-LE and BBS scores (p_a,b,c_ < 0.001) (Figure 2).

### 3.3 Local activation of brain networks

#### 3.3.1 Comparison of three groups of ALFF and ReHo before and after treatment

**Figure 3:**
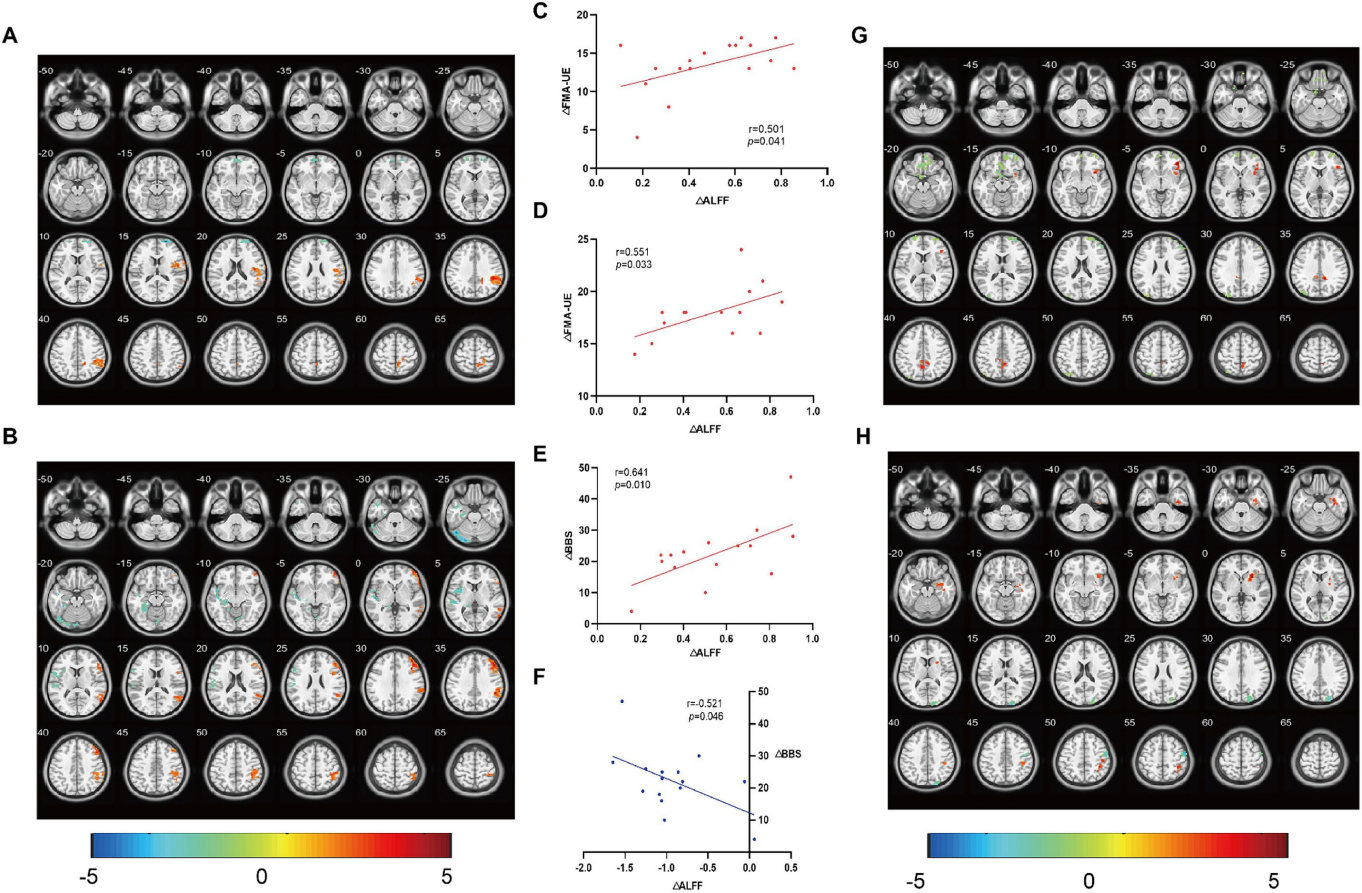
Comparison of ALFF/ReHo and correlation analysis. The changes of ALFF in brain regions before and after treatment and their correlation with clinical evaluation were analyzed. **(A,G)** brain regions with ALFF and ReHo changes in M1 group, and **(B,H)** brain regions with ALFF and ReHo changes in SMA group. **(C)** The change of ALFF in the right supramargial gyrus (affected side) was positively correlated with the change of FMA-UE in the M1 group, **(D)** the change of ALFF in the right supramargial gyrus (affected side) was positively correlated with the change of FMA-UE in the SMA group, and **(E)** the increase of ALFF in the right middle temporal gyrus (affected side) was positively correlated with the improvement of BBS in the SMA group. **(F)** There was a negative correlation between the change of ALFF in the left (contralateral) cerebellum and the improvement of BBS in the SMA group. The color bar represents the intensity of ALFF/ReHo changes in a given brain area.

Compared ALFF before and after treatment, the M1 group showed an increase in the right superior marginal gyrus, anterior cuneiform lobe, and postcentral gyrus. The difference between the right superior marginal gyrus and anterior cuneiform lobe was statistically significant (q_FDR corr._ < 0.05); the left rectus gyri, the right dorsolateral superior frontal gyrus, and the middle occipital gyrus decreased, with significant differences in the right dorsolateral superior frontal gyrus (q_FDR corr._ < 0.05) (Figure 3A). The SMA group showed an increase in the right middle temporal gyrus, postcentral gyrus, inferior parietal angular gyrus, middle frontal gyrus, precentral gyrus, left anterior cuneiform lobe, and left and right supplementary motor areas, with significant differences in the right middle temporal gyrus, inferior parietal angular gyrus, and middle frontal gyrus (q_FDR corr._ < 0.05);There was a decrease in the left cerebellum, superior temporal gyrus, precentral gyrus, postcentral gyrus, fusiform gyrus, insula, cortex around the right calcarine fissure, and the right anterior cingulate and paracingulate gyrus, with significant differences in the left cerebellum, superior temporal gyrus, fusiform gyrus, and insula (q_FDR corr._ < 0.05) (Figure 3B). No significant changes were found in the brain regions of the Sham group. The change of ALFF in the right supramargial gyrus (affected side) was positively correlated with the change of FMA-UE in the M1 group (Figure 3C) and SMA group (Figure 3D); and the increase of ALFF in the right middle temporal gyrus (affected side) was positively correlated with the improvement of BBS in the SMA group (Figure 3E). There was a negative correlation between the change of ALFF in the left (contralateral) cerebellum and the improvement of BBS in the SMA group (Figure 3F).

Compared ReHo before and after treatment, the M1 group showed an increase in the left and right cerebellum, right insula, supramarginal gyrus, anterior cuneiform gyrus, and left inferior parietal angular gyrus. Among them, the difference between the right insula and anterior cuneiform gyrus was significant (q_FDR corr._ < 0.05); The right inferior temporal gyrus, left rectus gyrus, right superior frontal gyrus, and left postcentral gyrus decreased, while the left rectus gyrus and right superior frontal gyrus showed significant differences compared to before (q_FDR corr._ < 0.05) (Figure 3G); The SMA group showed an increase in the right hippocampus, putamen, insular inferior frontal gyrus, inferior parietal lobule, precentral gyrus, postcentral gyrus, left and right supplementary motor area, left middle frontal gyrus, and anterior cuneus, with a significant increase in the right hippocampus, putamen, and inferior parietal lobule (q_FDR corr._ < 0.05); The left cerebellum and right occipital gyrus decreased, while the difference in the right occipital gyrus was significant (q_FDR corr._ < 0.05) (Figure 3H). No significant differences were found in the Sham group. No correlation was found between ReHo changes and functional improvement.

#### 3.3.2 Comparison of ALFF and ReHo values among the M1 group, SMA group, and Sham group after treatment

**Figure 4:**
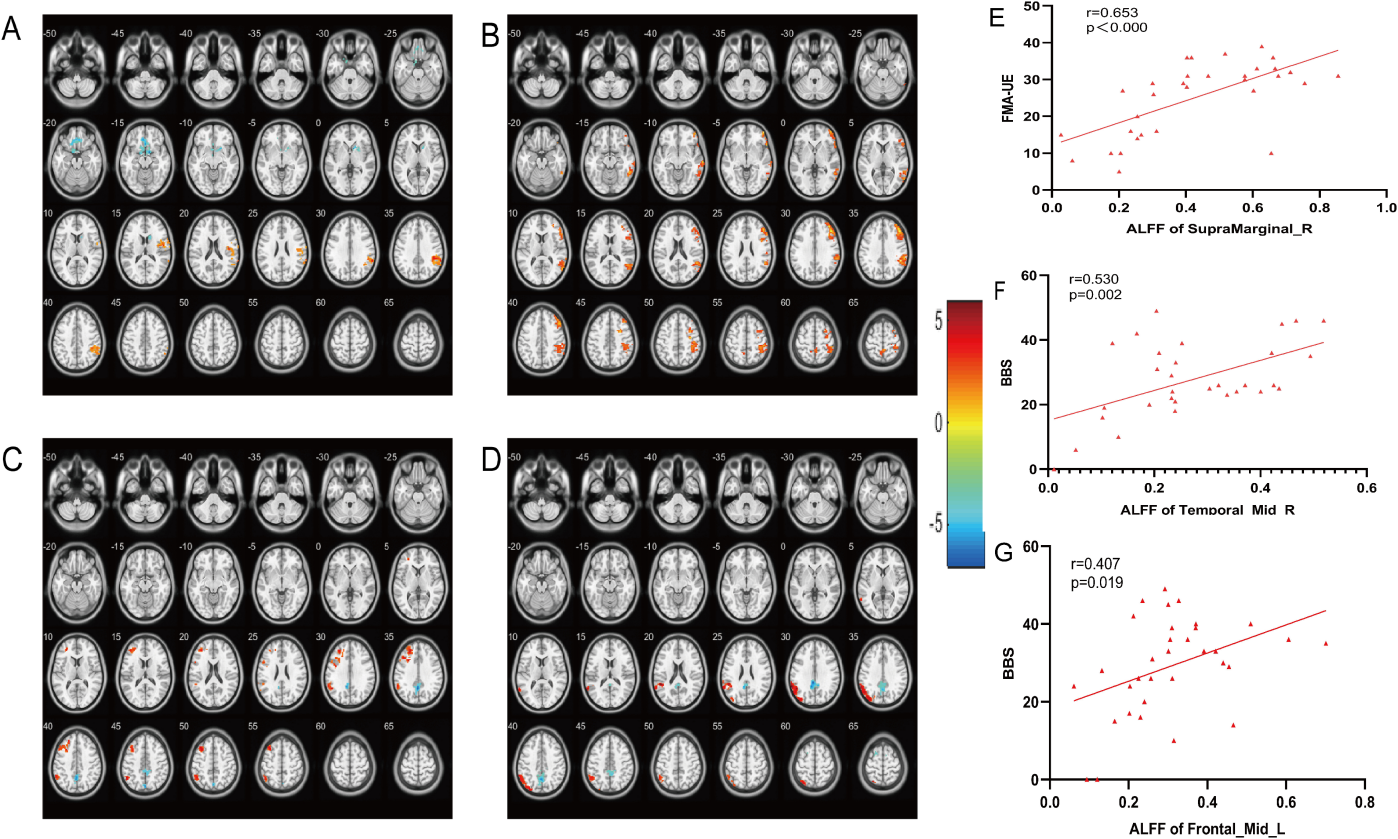
Comparison of brain regions with changes in ALFF and ReHo among the three groups after treatment. **(A)** Changing Brain changes in ALFF after treatment in (M1 group vs. Sham group). **(B)** Changing Brain changes in ALFF after treatment in (SMA group vs. Sham group). **(C)** Changing Brain changes in ALFF after treatment in (M1 group vs. SMA group). **(D)** Changing Brain changes in ReHo after treatment in (M1 group vs. SMA group). The color bar on the right represents the activation intensity of brain regions. **(E–G)** Correlation analysis between ALFF changes in brain regions and motor function scores.

The ALFF in the M1 group was significantly higher in the right marginal gyrus than in the Sham group (Figure 4A); the SMA group showed significant elevation in the right middle temporal gyrus (Figure 4B). After treatment, compared with the M1 group, the SMA group showed a significant decrease in ALFF in the left Precuneus, while the left superior marginal gyrus and middle frontal gyrus showed a significant increase (Figure 4C), and ReHo decreased in the cingulate gyrus and increased in the left parietal lobe and angular gyrus (Figure 4D) (*post hoc t* test, *p* < 0.05, FDR corrected at cluster level). Correlation analysis showed that the ALFF of the right supramarginal gyrus was positively correlated with FMA-UE (M1 group and Sham group, *r* = 0.653, *p* < 0.000, Figure 4E), the right middle temporal gyrus was positively correlated with BBS (SMA group and Sham group, *r* = 0.530, *p* = 0.002, Figure 4F), and the left middle frontal gyrus was positively correlated with BBS (M1group and SMA group, *r* = 0.407, *p* = 0.019, Figure 4G).

### 3.4 Functional connection

**Figure 5:**
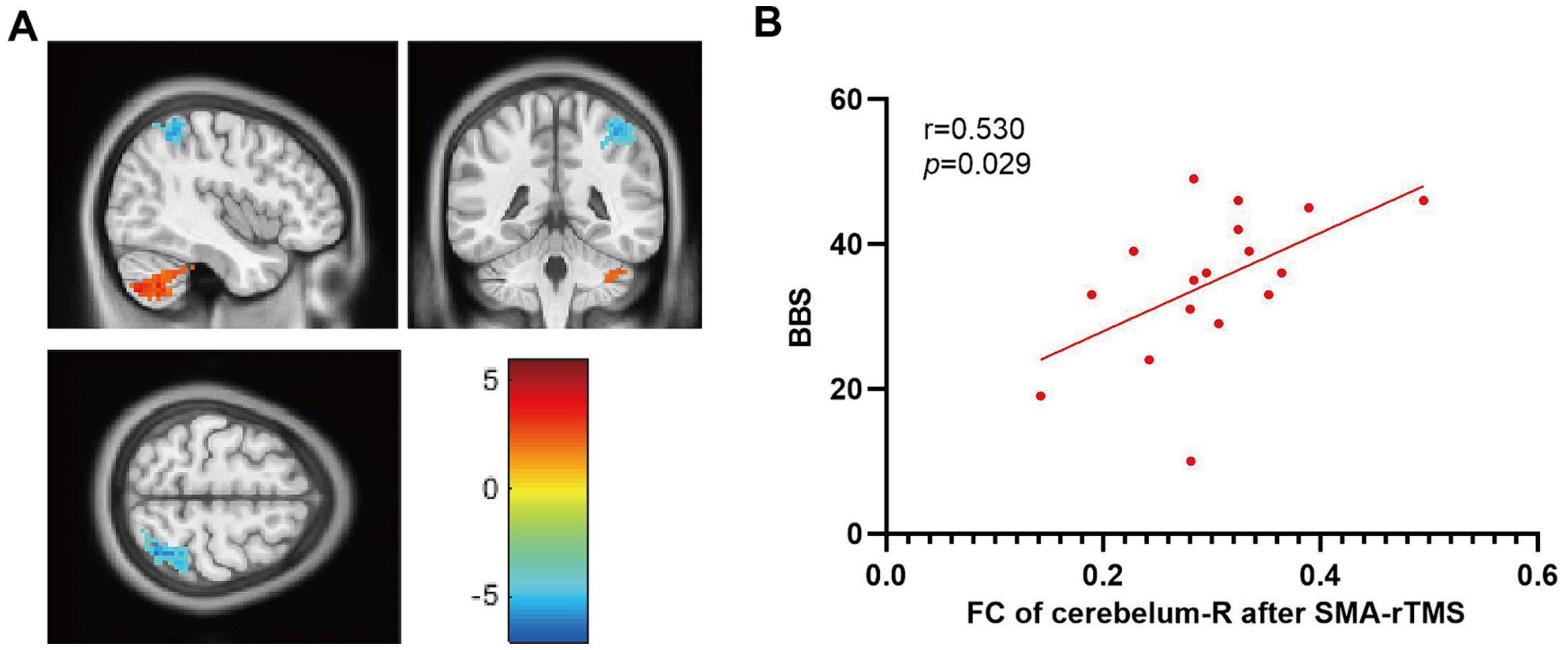
Comparison of functional connectivity between the M1 and SMA groups after treatment. **(A)** FC changes in brain regions, **(B)** Correlation between FC changes and BBS. The color bar on the right represents the intensity of brain activation.

After treatment, the functional connection between the right supplementary motor area and the right cerebellar hemisphere in the SMA group was enhanced compared to that in the M1 group (voxel: 96, T = 5.0161, q_FDR corr._ < 0.05); FC in the ipsilateral parietal lobe weakened (voxel: 97, T = -4.1696, q_FDR corr._ < 0.05). Correlational analysis showed that changes in resting-state functional connectivity between the right supplementary motor area and the right cerebellum were correlated with improvements in BBS scores (*r* = 0.530, *p* = 0.029) (Figure 5). No significant correlation was observed between resting-state functional connectivity and other clinical evaluations.

## 4 Discussion

This randomized, double-blind, controlled clinical trial is the first to systematically compare the effects of high-frequency rTMS targeting the M1 and SMA in the affected cerebral hemisphere on motor function after stroke. Our results showed significant improvements in limb motor function (FMA-UE and FMA-LE scores) and balance function (BBS) in all three groups. Our findings further demonstrate that SMA-rTMS significantly enhances motor function in patients, encompassing improvements in limb movement and balance, when compared to the Sham group. Notably, the recovery of balance function following 4 weeks of SMA-rTMS intervention is markedly superior to that observed with M1-rTMS. Additionally, we identified a positive correlation between local activation in the middle temporal gyrus (SMA and Sham groups) and middle frontal gyrus (M1 and SMA groups) with the recovery of balance function; conversely, local activation in the contralateral cerebellum (SMA group) was found to hinder this recovery. The functional connectivity between SMA and the ipsilateral cerebellum (SMA group) appears to facilitate balance recovery. Furthermore, local activation within the supramarginal gyrus (three groups) positively correlates with enhancements in upper limb functionality.

### 4.1 Effect of rTMS on clinical motor function in stroke patients

Research has shown (29–32)that TMS has a significant effect on the improvement of motor function after stroke; for example, it can significantly improve the recovery of motor functions such as upper and lower limb movement, balance function, and gait after stroke. This study obtained similar results, with significant improvements in limb movement (FMA-UE, FMA-LE) and balance function (BBS) in stroke patients after high-frequency (10 Hz) rTMS intervention compared to the control group and before intervention. This study is the first to conduct a randomized controlled study on different targets (M1, SMA), The results showed that compared to the sham rTMS, both M1-rTMS and SMA-rTMS significantly improved motor function and balance measured by FM and BBS. Further, SMA-rTMS led to similar improvement on motor function but significantly greater improvement on balance compared to M1-rTMS.

### 4.2 Clinical function and brain activity

rs-fMRI is widely used and has become a source of biomarkers for the study of complex brain networks after stroke (33–36). ALFF (Amplitude of Low Frequency Fluctuations) is a method to measure local spontaneous brain activity, which reflects the activity intensity of local brain regions by analyzing the amplitude of low-frequency oscillation signals in gray matter at rest. ALFF mainly focuses on the amplitude of low-frequency oscillations in voxels, reflecting the local spontaneous activity in the resting state. In addition, ReHo (Regional Homogeneity) is a method to measure the homogeneity of neural activity in a local brain region, which evaluates the activity pattern of a local brain region by analyzing the similarity of neural activity between adjacent voxels. ReHo reflects the regional homogeneity of neural activity between adjacent voxels and is often used to assess concordant activity in local brain regions. Previous studies (19, 37, 38) have found that the excitability (ALFF, ReHo) of the lesion hemisphere after stroke was significantly reduced compared to that of healthy controls, while that of the contralateral hemisphere was significantly increased. This may be due to the delayed effects of injury on the affected hemisphere, which leads to a reduction in the inhibitory effect of the affected hemisphere on the healthy hemisphere and the enhancement of excitability on the healthy side. In turn, the excessive inhibitory effect of the healthy side on the affected side via the corpus callosum further aggravates the injury of the affected side (39, 40). After rTMS intervention, we observed that the excitability of the affected hemisphere was significantly increased and that of the contralateral hemisphere was decreased, indicating that rTMS played an important role in regulating the functional balance of the cerebral hemisphere.

On this basis, we conducted a correlation study between brain area activation and motor function score and found that there was a negative correlation between the changes in the activation level of the contralateral cerebellum (ALFF) and balance function score (BBS) after stroke. We hypothesized that too strong activation of unilateral cerebellum may be detrimental to the recovery of balance function after stroke. Considering that balance is dominated by the bilateral cerebellum, the activation of the bilateral cerebellum tending to a steady state may be more conducive to the recovery of balance function. The activation level of the affected cerebellum was not significantly enhanced compared with before, but the activation level of the whole affected hemisphere was generally increased after the intervention. It is speculated that the affected cerebellum may not show significant changes due to the problem of sample size. In the future, we will expand the sample size to continue studying this interesting finding.

In addition, the activation and retention (ALFF) of the middle temporal gyrus on the affected side was positively correlated with balance function (BBS), suggesting the role of the middle temporal gyrus in balance function. The temporal lobe is surrounded by the frontal, parietal, and occipital lobes and has extensive connections with other brain regions, making its functions complex. The middle temporal gyrus is located in the middle of the temporal lobe and plays an important role in brain function. Some studies have shown (41) that the middle temporal gyrus has a greater impact on trunk ataxia, and this study also confirmed that the retention of middle temporal gyrus function has an impact on balance function.

The present study found significant activation of the supramarginal gyrus on the affected side in the M1, SMA and Sham groups, and it was found to correlate with the degree of recovery of upper limb dysfunction. The supramarginal gyrus is located in the front of the inferior parietal lobule. Some studies have shown that (42, 43) after TMS intervention, the local activity of the parietal cortex is related to improvements in motor function and cerebral cortex reorganization after stroke. The supramarginal gyrus, located in the secondary somatosensory cortex, responds to physical stimuli and completes the task of structural differentiation. It is an important center responsible for fine motor coordination as well as complex athletic and occupational skills (42, 44). This study also confirmed its correlation with upper limb movement. In addition, the closer connection between the SMA and other brain regions has also been confirmed. Correlation analysis revealed that there was a positive correlation between the activity of the affected temporal lobe nerve and the preservation of balance function after stroke. After 10 Hz rTMS intervention on the affected side, the same results were found in the SMA group. The enhancement of neural activity in the cerebellum and middle temporal gyrus was positively correlated with the balance function score.

### 4.3 Clinical function and brain functional connectivity

Seed-based FC (seed-based FC) is a common method for functional connectivity analysis, which can be used to explain the functional connectivity correlation between the brain area of interest and other regions of the brain (45, 46). After intervention, the SMA group showed a significant increase in functional connectivity between the affected SMA brain area and the ipsilesional cerebellum compared to the M1 group, and it was found to be positively correlated with the recovery of balance function (BBS). This indicates that SMA-targeted rTMS stimulation can regulate the coupling between the SMA and the ipsilateral cerebellum and once again confirms that the cerebellum is more closely connected to the SMA than to M1.

The reorganization of brain function after rTMS intervention is a key step in rehabilitation (47). In this analysis of resting state functional connectivity, we observed a significant enhancement in SMA cerebellar functional connectivity, which is correlated with improvement in balance function. In theory, there is also a widespread association between M1 and cerebellar functional connectivity, but unfortunately, we did not observe this phenomenon in this study. Considering that SMA and cerebellum may have a broader connection through the cortical pontine cerebellar tract or cerebellar thalamic cortical pathway (48, 49). M1 may mainly affect motor function through the corticospinal tract, involving limb muscle strength and endurance. The FC between M1 and cerebellum showed an increasing trend in this study, but no significant difference was found. The sample size will be further expanded to verify this result.

### 4.4 Limitations of this study

Due to limited time and the COVID-19 the collected samples are not large enough, and the results may be biased. In addition, the image reversal process may have allowed lateralized features of left and right hemisphere stroke injuries to go unnoticed. Therefore, in future research, we need to compare studies conducted on diverse scales, at various centers, and with multiple time points to verify or correct the results of the present study.

## 5 Conclusion

Our findings demonstrate that SMA-rTMS significantly enhances motor function in patients, encompassing improvements in limb movement and balance, when compared to the Sham group. Notably, the recovery of balance function following 4 weeks of SMA-rTMS intervention is markedly superior to that observed with M1-rTMS. Therefore, SMA-rTMS may provide a new way for the rehabilitation of motor function in patients after stroke.

## Data Availability

The raw data supporting the conclusions of this article will be made available by the authors, without undue reservation.

## Footnotes

^1^ http://rfmri.org/dpabi
