# Supplementary material for: Effects of repetitive transcranial magnetic stimulation at different targets on brain function in stroke patients: a randomized controlled trial
Source: Front Neurol. 2024 Sep 30;15:1454220. doi: 10.3389/fneur.2024.1454220 (PMC11471684; doi:10.3389/fneur.2024.1454220)
Supplement: Supplementary file 1 [file Table_1.DOCX]

**Supplemental Material 1**

**Table 1 Seed Point Coordinates**

| ID | Region | Side | Abbreviation | MNI Coordinate | | | Radius |
| --- | --- | --- | --- | --- | --- | --- | --- |
|  |  |  |  | X | Y | Z |  |
| 1 | Primary motor cortex | L | M1.L | -38 | -22 | 56 | 6 |
| 2 | Primary motor cortex | R | M1.R | 38 | -22 | 56 | 6 |
| 3 | Supplementary motor area | L | SMA.L | -5 | -4 | 57 | 6 |
| 4 | Supplementary motor area | R | SMA.R | 5 | -4 | 57 | 6 |

**Supplemental Material 2**

**Table 3 Comparison of ALFF values among the three groups before and after treatment**

| Contrast  （M1） | Structure | voxels | | Peak MNI | T | | Contrast  (SMA) | Structure | voxels | Peak MNI | T |
| --- | --- | --- | --- | --- | --- | --- | --- | --- | --- | --- | --- |
| Post＞Pre | SupraMarginal_R | | 151 | 39 -9 18 | | 6.1664 | Post＞Pre | Temporal_Mid_R | 139 | 60 -42 3 | 4.6726 |
|  | Precuneus_R | | 81 | 18 -42 36 | | 4.7215 |  | SupraMarginal_R | 121 | 51 -25 36 | 7.591 |
|  |  | |  |  | |  |  | Postcentral_R | 92 |  |  |
|  |  | |  |  | |  |  | Frontal_Mid_R | 171 | 48 30 33 | 6.3858 |
|  |  | |  |  | |  |  | Parietal_Inf_R | 94 |  |  |
| Post＜Pre | Frontal_Sup_R | | 71 | 21 63 15 | | -5.6192 | Post＜Pre | Cerebelum_Crus1_L | 134 | -45 -66 -24 | -6.6659 |
|  |  | |  |  | |  |  | Temporal_Sup_L | 110 | -42 -30 3 | -7.0761 |
|  |  | |  |  | |  |  | Fusiform_L | 70 | -33 -48 -12 | -5.5679 |
|  |  | |  |  | |  |  | Insula_L | 42 | -39 3 3 | -6.0761 |

**Table 4 Comparison of ReHo before and after treatment in the three groups**

| Contrast  （M1） | Structure | voxels | Peak MNI | T | Contrast  (SMA) | Structure | voxels | Peak MNI | T |
| --- | --- | --- | --- | --- | --- | --- | --- | --- | --- |
| Post＞Pre | Insula_R | 43 | 39 36 -6 | 7.0136 | Post＞Pre | Hippocampus_R | 40 | 36 -18 -24 | 4.24 |
|  | Precuneus_R | 71 | 12 -45 36 | 6.2393 |  | Putamen_R | 53 | 27 15 0 | 6.6447 |
|  |  |  |  |  |  | Parietal_Inf_R | 45 | 45 -24 48 | 5.2273 |
| Post＜Pre | Rectus_L | 72 | -3 6 -15 | -5.9781 | Post＜Pre | Occipital_Sup_R | 142 | 15 -102 12 | -7.5944 |
|  | Frontal_Sup_R | 53 | 36 57 18 | -8.8241 |  |  |  |  |  |

**Table 5 the functional connectivity of right SMA seed points**

| Contrast | Structure | voxels | Peak MNI | T | Contrast | Structure | voxels | Peak MNI | T |
| --- | --- | --- | --- | --- | --- | --- | --- | --- | --- |
| + | Cerebelum_Crus2_R | 96 | 45 -51 -48 | 5.0161 | - | Parietal_Sup_R | 97 | 36 -57 57 | -4.1696 |
